# Disambiguation of ambiguous figures in the brain

**DOI:** 10.3389/fnhum.2013.00501

**Published:** 2013-08-30

**Authors:** Tomohiro Ishizu

**Affiliations:** ^1^Faculty of Letters, Arts and Sciences, School of Humanities and Social Sciences, Waseda UniversityTokyo, Japan; ^2^Japan Society for the Promotion of ScienceTokyo, Japan; ^3^Department of Cell and Developmental Biology, Wellcome Laboratory of Neurobiology, University College LondonLondon, UK

**Keywords:** magnetoencephalography, prior knowledge, expectation, body perception, visual perception

## Abstract

Disambiguation refers to the ability to interpret ambiguous information in a sensible way, which is important in an ever-changing external environment. Disambiguation occurs when prior knowledge is given before an ambiguous stimulus is presented. For example, labeling a series of meaningless blobs as a “human body” can change the observer's perception. The aim of this experiment was to study the neural circuitry underlying disambiguation caused by prior knowledge. We presented to participants a series of meaningless blobs with different contextual information. As participants performed this task, we used magnetoencephalography to map the brain areas that were activated when participants perceived blobs as a human body. The participants were presented identical sets of blob stimuli, and were instructed that a human body would appear more frequently in the “high body” condition than in the “low body” condition. We found the blob stimuli were more frequently perceived as the human body when they were presented in the “high body” condition. Such contextual modulation correlated with activity in the extrastriate body area (EBA) and the inferior frontal gyrus (IFG). Furthermore, we observed that IFG activation preceded EBA activation. These findings suggest that top-down processing in the IFG plays a role in disambiguating ambiguous information and modifying an individual's perceptions.

“We don't see things as they are, we see them as we are”—Anaïs Nin

## Introduction

Disambiguation is the ability to interpret ambiguous information in a sensible way, and it is considered one of the fundamental components of the creativity process (Riquelme, 2002; Wiseman et al., 2011). This is true in terms of art appreciation and when creating works of art (Zeki, 2008). Many art masterpieces contain ambiguity that requires disambiguation for their interpretation. “*Girl with a pearl earring*” by Johannes Vermeer, “*Pietà of Rondanini*” by Michelangelo, Escher's bi-stable drawings, and Gestalt images are all examples of images that require disambiguation. When the same person views Vermeer's portrait at different times or in different situations, their perceptions of the girl's facial expression in the painting can change. For example, they may perceive the girl's sentiment as pleasure one day, but as sadness, innocence, or seduction on a different day. Gestalt images, which initially might appear as a collection of ambiguous blobs, can become a meaningful figure through disambiguation.

Disambiguation is important in the art world, but it also speaks to our daily experiences. Our perception of the external world is dynamic, and interpreting the information in our environment can be complicated when the external world is ever-changing (Zeki, 2008). Disambiguation was demonstrated in previous studies in which subjects were influenced by prior knowledge. In these studies, prior knowledge indicated that one potential interpretation had a greater likelihood than another interpretation. As a result, subjects often made biased decisions in favor of the indicated alternative (Green and Swets, 1966). Psychophysical studies have shown that prior knowledge can influence how we perceive emotions through facial expressions (Wallbott, 1988; Kawin, 1992), facilitate recognition of stimuli that are otherwise imperceptible (Cox et al., 2004), and alter how we perceive objects, even when the stimulus remains physically unchanged (Bentin and Golland, 2002). These psychophysical studies suggest that prior knowledge alters perception and interpretation by producing expectations for a stimulus, which is presumably mediated by a “top-down” influence (Bar, 2004; Mobbs et al., 2006). Neuroimaging studies indicate that several brain regions are recruited during visual disambiguation with prior knowledge, including the prefrontal and parietal cortical areas (Mobbs et al., 2006; Hansen et al., 2012), and the visual areas relevant to the stimulus (Andrews and Schluppeck, 2004). Although the frontal regions likely play a major role in this top-down process (Mobbs et al., 2006), the neural mechanisms underlying disambiguation, and the functional relationships among the brain areas mediating such mechanisms, are poorly understood.

Event-related potential (ERP) studies indicate the N170 component is associated with face perception (Bentin et al., 1999). Neuroimaging studies that investigated face perception indicated that stimuli such as realistic minute figures or ambiguous face figures could induce face-selective neural responses, but the N170 component is remarkably diminished in participants who fail to recognize a face in such stimuli. This implies the face-selective N170 is strongly linked with both low-level perceptual features and conscious face recognition (Bentin and Golland, 2002). These ambiguous and functionally specialized visual stimuli are especially useful in investigating the neural systems that participate in object disambiguation.

Body-selective regions also have been identified in the brain. One such area, the extrastriate body area (EBA), produces an ERP and event-related magnetic field (ERF) that is similar to the N170 (Thierry et al., 2006; Ishizu et al., 2010). Body figures, however, are more easily portrayed with ambiguity than face figures because they have less salience than facial images. Therefore, in the present study, we investigated the neural circuitry underlying disambiguation of ambiguous body figure stimuli when subjects received prior knowledge about the stimuli. We presented meaningless blobs to subjects and used magnetoencephalography (MEG) to map the brain areas that were active when subjects perceived the blobs with varying levels of prior knowledge that indicated the blobs represented body images.

## Methods

### Subjects

Twelve healthy, right-handed volunteers (6 males, 6 females; mean age, 28.8 years) participated in this study. Participants had normal or corrected-to-normal vision, and no participant had a history of neurological or psychiatric disorder. This study was approved by the Ethics Committee of Keio University (Tokyo, Japan) and The Code of Ethics of the World Medical Association (Declaration of Helsinki; printed in the British Medical Journal, 18 July 1964). All participants provided their written informed consent before participating in this study. Data were anonymized.

### Psychophysical testing and stimuli

Prior to the MEG experiment, psychophysical tests were conducted to select the stimuli. In these tests, subjects performed a two-alternative forced choice task in which they judged whether a stimulus was a body or a collection of blobs. We generated 300 black and white blob images by using image-editing software (Adobe® Photoshop CS3®, Mountain View, CA, USA). Of these 300 blob images, 250 were considered meaningless blobs by 20 naïve observers who did not participate in the experiment (10 males, 10 females; mean age, 25.3 years).

**Figure 1:**
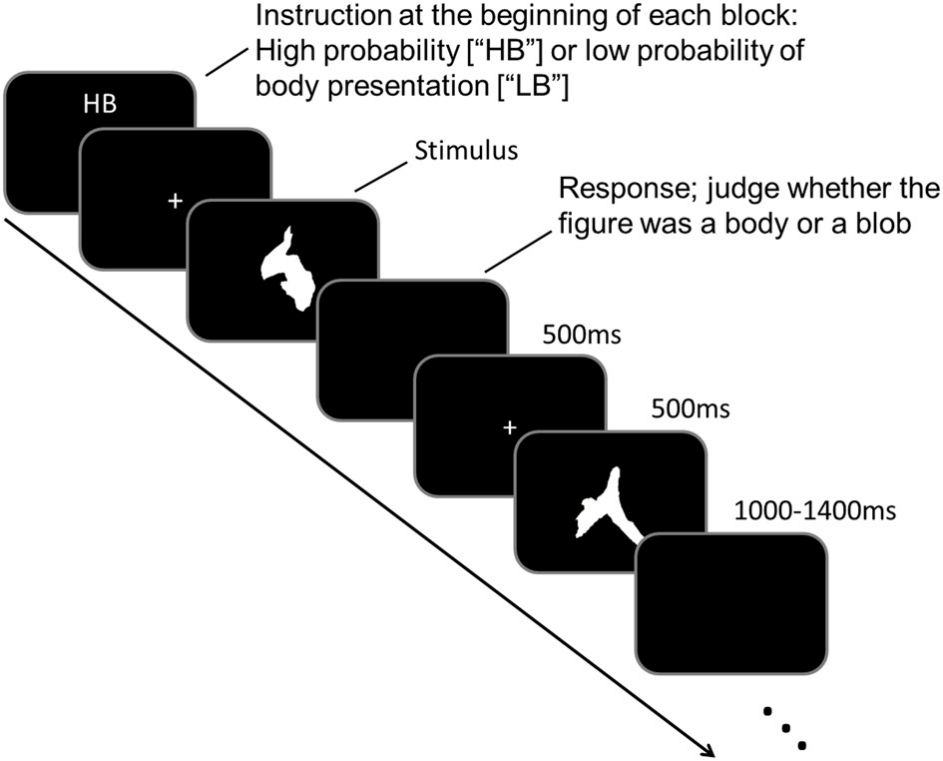
**An illustration of the paradigm and examples of the stimuli used in this study**.

As participants underwent MEG scanning, they performed a task in which they judged the 250 meaningless blob images. Participants were asked to judge whether a blob image was a body figure. Prior to MEG scanning, participants were instructed that there were two experimental conditions: “high body” (HB) and “low body” (LB) conditions. In the HB condition, participants were instructed that body figures would be presented randomly in 50–70% of the images, whereas in the LB condition, they were instructed that body figures would be presented randomly in 10–30% of the images. Identical images were used for body and blob presentations, which prevented physical differences among the images from confounding the results. The experiment consisted of ten blocks, with five blocks performed for each condition, and each block consisted of 50 trials. An illustration of the experimental paradigm is shown in Figure 1.

Each block began when an “HB” or “LB” was presented on a black screen (<0.2 cd/m^2^) for 4 s, which indicated the task condition to the participant. Each trial began with a white fixation point (“+” sign; 0.1° × 0.1°) that was presented in the middle of the black screen for 500 ms. The fixation point was followed by a target stimulus that was presented for 500 ms. The target stimulus was a blob image, and participants had to judge whether a body figure was represented in the blob image. Participants responded by pressing the appropriate button during the intertrial interval (ITI). The ITI varied randomly between 1000 and 1400 ms. The task sequence was counterbalanced across participants.

Behavioral data were analyzed using a two-tailed paired *t*-test. We compared the number of trials in which blobs were perceived as body figures between the HB and LB conditions. The image stimuli were sequenced using STIM2 (Neuroscan, Texas, USA) and subtended to a maximum of 4° × 5° of the visual angle. The presentation order was randomized across participants.

### MEG recordings

MEG scans were performed in a magnetically shielded room at the University of Tokyo Hospital by using a 306-channel whole-scalp neuromagnetometer (Vectorview; Neuromag, Helsinki, Finland). Participants sat in a reclining chair as MEG signals were recorded with 204 planar-type gradiometers. As previously reported (Nishitani and Hari, 2000), these planar sensors provide the strongest signals when they are located just above the targeted area of the cerebral cortex. The event trigger was synchronized to the onset of stimulus presentation. The MEG signals were band-pass filtered at 0.01–100 Hz, sampled at 1000 Hz, and stored for offline analysis. The averaging and analysis period was from −100 to 500 ms time-locked to the onset of a stimulus. Trials without responses or those contaminated with eye blinking or body movements (MEG amplitude >4000 fT/cm) were excluded from the averaging. Eye blinks were recorded with electrodes placed above and below the right eye, and on the outer canthi of the right and left eyes (vertical and horizontal electrooculogram amplitude >150 μ V). At least 180 epochs were recorded in each test condition for averaging (average = 220; *SD* = 18.2).

Prior to performing the MEG recordings, four head-position-indicator (HPI) coils were placed on the scalp. The sensor placement was determined by measuring the magnetic signals produced by weak currents that led into the four indicator coils. For the alignment of the MEG and magnetic resonance imaging (MRI) coordinate systems, we determined the coil locations relative to specific anatomical landmarks (nasion and bilateral preauricular points) using a 3D digitizer (Isotrak; Polhemus, Colchester, Vermont, USA). Head-system MR images were obtained using a 3.0-T MRI system (Trio Tim; Siemens, Erlangen, Germany). Signals recorded from the 204 planar gradiometers measured two orthogonal derivatives of the radial magnetic field, which amounted to 102 locations on the head.

### MEG analysis

#### Latencies and amplitudes of MEG components

The averaged data were digitally filtered offline at 0.5–30 Hz. A 100-ms pre-stimulus baseline was used to evaluate responses to the stimuli. We then determined the differences between MEG signals for the events in which a body was perceived and the events in which a blob was perceived. In order to test the disambiguation effect, and to avoid differences in low-level visual features, we analyzed data only from trials in which participants reported a body in the HB condition and a blob in the LB condition in response to *the same* stimulus. This resulted in at least 100 trials for each condition (average = 129; *SD* = 16.3).

Considering the MEG recording system measured a magnetic field gradient at a given location through a pair of gradiometers that were oriented perpendicular to each other, the gradient vector strength at each location was calculated using the data from each pair of gradiometers. Cortical activation is best measured at locations that show the largest deflections in the magnetic field gradient (Nishitani and Hari, 2000). For this reason, we used the channel pair that portrayed the largest deflection as the essential sensor for the response (ESR) component (Ayabe et al., 2008), and to determine the peak latency and amplitude for a given cortical location. Significant deflections were components that surpassed two standard deviations (SDs) of the baseline mean, and had a duration of at least 40 ms. We identified in all participants significant deflections that peaked at approximately 115 (90–120 ms), 135, and 187 ms (160–200 ms) after stimulus onset. These deflections were recorded in the occipital, occipito-temporal, and lateral frontal cortical areas. Peak latency was defined as the time interval from stimulus onset to the peak of the component. The onset and amplitude of the components were assessed for each participant across different channels and within the same cortical areas. The peak latencies and amplitudes for each MEG response, the hemisphere from which the response was recorded (left or right), and their associations with each category (blob or body), were analyzed using repeated-measures analysis of variance (ANOVA). If the assumption of sphericity was violated in Mauchly's sphericity test, the Greenhouse-Geisser correction coefficient epsilon was used to correct the degree of freedom.

### Source analysis

Source estimation was performed for each subject by using the multivariate source pre-localization method (MSP) (Mattout et al., 2005; Friston et al., 2008) in SPM8 (http://www.fil.ion.ucl.ac.uk/spm/software/spm8/). Gaussian random field theory was used to control for multiple comparisons in 3D space (source space; Kiebel and Friston, 2002). The main response for each component was estimated as the latency obtained for each subject.

Source localization took place within a time window of ±15 ms around the peak time. This criterion was established individually for each condition and for each subject. Source images for each condition and each response were smoothed using a Gaussian smoothing kernel of 8 × 8 × 8 mm, and taken to the second level (between subjects). Statistical maps were made for each condition (vs. baseline) using a one-sample *t*-test, and between two conditions (blob vs. body) using a paired *t*-test. The coordinates reported are the Montreal Neurological Institute (MNI) coordinates from the SPM output.

The source locations for the peak activation levels are reported with a significance threshold of *p*_(unc.)_ < 0.001. Although we report uncorrected statistics below, the statistical significance of ERFs were established by the latency analysis. Thus, the lower statistical threshold only applies to the locations where the peaks were recorded, not the existence of the responses. We identified the brain region where each peak was recorded using the SPM Anatomy toolbox (http://www2.fz-juelich.de/inm/index.php?index=194). If multiple peaks were present within an area, then the peak with the greatest amplitude was chosen for analysis.

## Results

### Behavioral results

The behavioral responses recorded during the LB and HB conditions indicated that, on average, participants perceived the body in 67% (*SD* = 8.3) of the HB trials and in 26.7% (*SD* = 6.7) of the LB trials. The meaningless stimuli were more frequently perceived as a body when they were presented with the HB instruction than when they were presented with the LB instruction (Paired *t*-test, *t* = 13.5; *df* = 11; *p* < 0.001). This result demonstrates that the instruction significantly altered the observer's perception.

### MEG results

**Figure 2:**
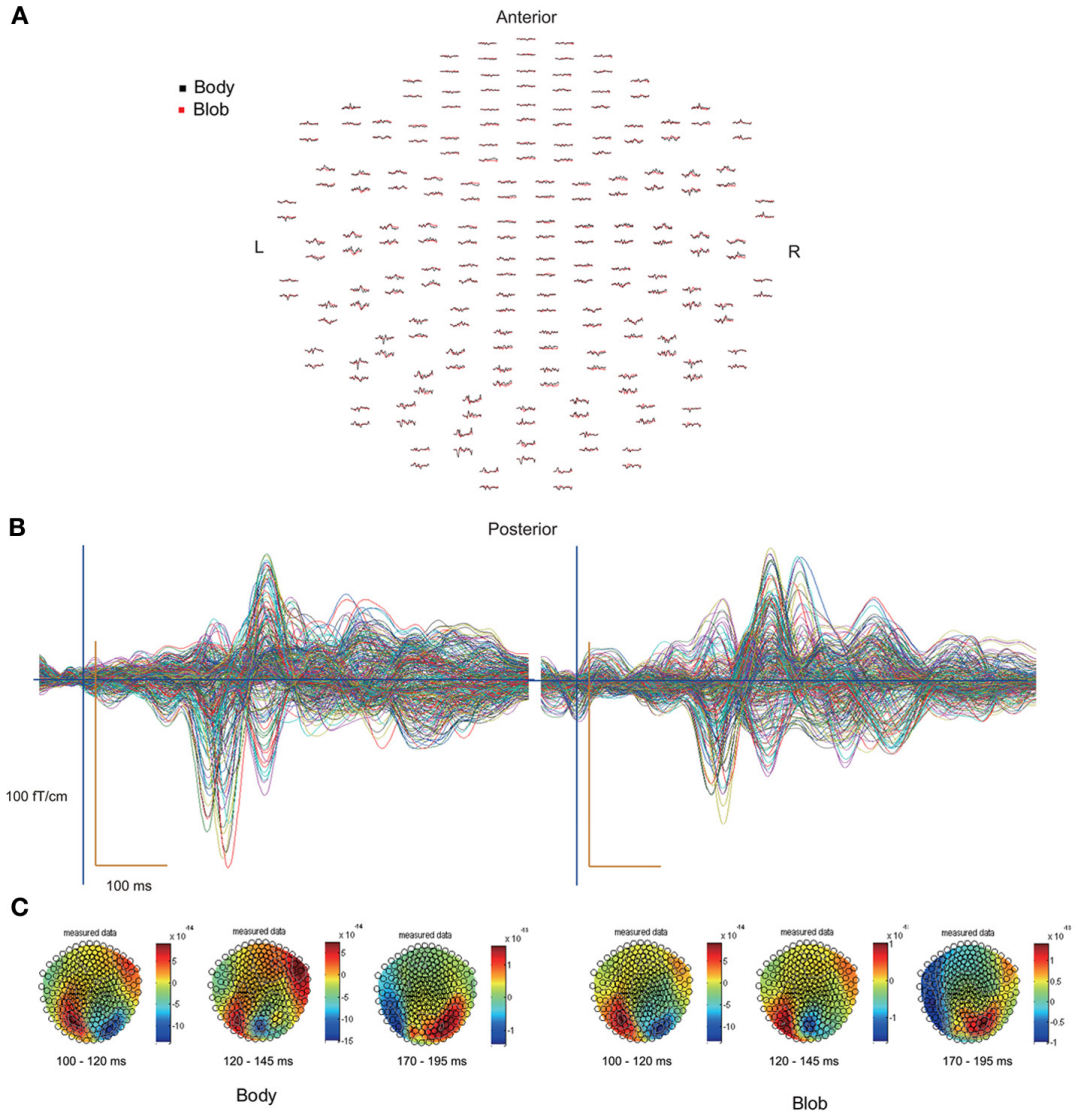
**(A)** Whole-scalp sensors waveforms (body, black line; blob, red line) and **(B)** super-imposed waveforms of the “body” and “blob” responses in a representative subject. **(C)** MEG contour maps at 115, 135, and 187 ms, respectively, after stimulus onsets.

**Figure 3:**
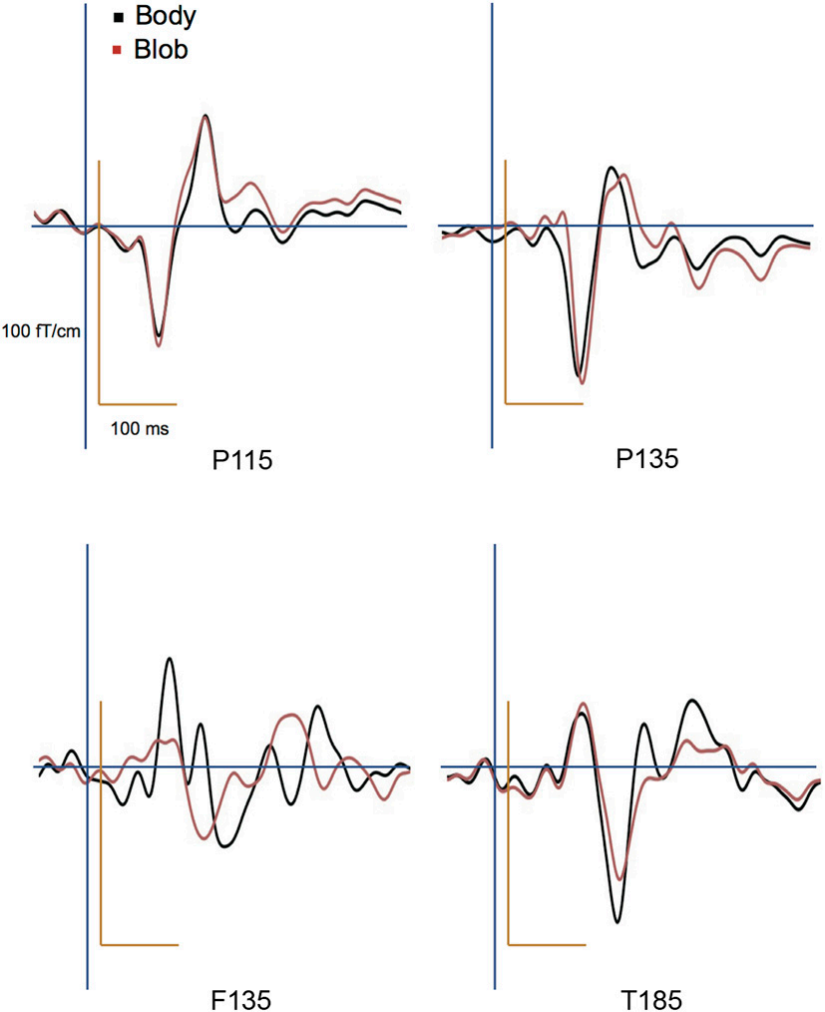
**Across-subjects averaged ERF waveforms in the right occipital, occipito-temporal, and front-temporal channels for “body” and “blob” responses (body, black line; blob, red line)**.

**Figure 4:**
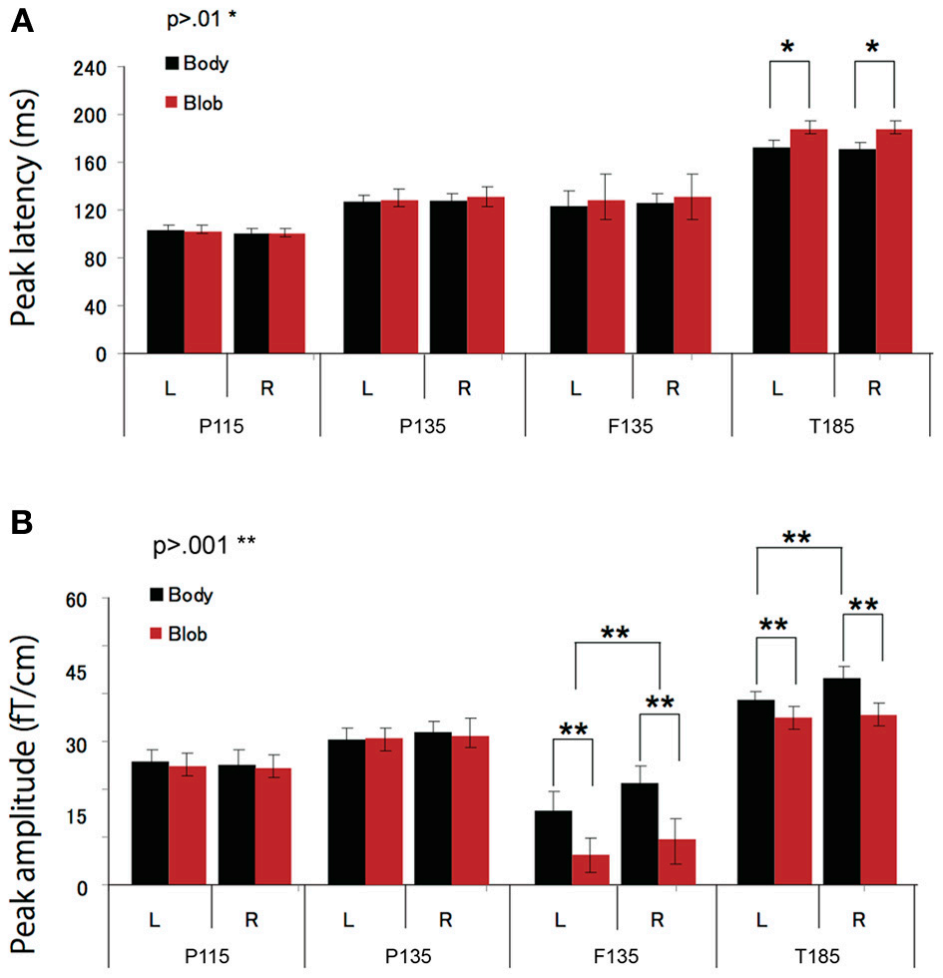
**Across-subjects mean (±SEM) of P115, P135, F135, and T185 peak latency (A) and amplitude (B) for “body” and “blob” responses**.

All participants showed prominent deflections bilaterally for both body and blob responses that showed average peak latencies at 115, 135, and 187 ms. Regarding the 135-ms component, prominent deflections were observed in the occipito-temporal area for both conditions, and in the frontal area for the body condition alone. The components were termed depending on the locations where they were recorded. P115 and P135 indicated posterior occipital area deflections, F135 indicated frontal area deflections, and T185 indicated occipito-temporal area deflections. Figures 2A,B show representative whole-scalp waveforms and the super-imposed waveforms of the body and blob responses. In these waveforms, clear deflections can be identified in each area at the latencies described above. Figure 2C shows the MEG contour maps for each peak (115, 135, and 187 ms) in which red lines indicate efflux from the head and blue lines indicate influx into the head. Again, clear influx-efflux patterns can be observed over occipital, temporal, and frontal areas. Figure 3 shows the across-subjects averaged ERF waveforms in the occipital, occipito-temporal, and front-temporal channels for body and blob responses. Figure 4 represents bar graphs of averaged ERF amplitudes and latencies for both response types for all subjects.

#### P115 response

Peak latency was observed on average at 115 ms, and was most prominent in the occipital regions. The average peak latencies were 116 ms for body responses and 115 ms for blob responses. Similarly, there was no significant main effect or interaction for the latency or amplitude of the P115 component.

#### P135 and F135 responses

We observed two clusters for body responses that were located in the occipito-temporal/parietal (P135) and frontal areas (F135). For blob responses, the component was prominent only in the occipito-temporal area. On average, the peak latency was observed at 134 ms for P135 and 137 ms for F135. Because the mean latency differed between the two components (*t*-test, *p* < 0.01), and the sensors that recorded each component were distinctly located within either the occipito-temporal or frontal areas, P135 and F135 were considered independent components. For P135, there was no significant main effect of category or hemisphere for amplitude or latency. On average, peak latencies at occipito-temporal/parietal sites for body responses were 135 ms, whereas those for blob responses were 136 ms. For F135, we observed that blob responses were rare. Therefore, we used the same time window to determine the latency and amplitude of body and blob responses. For amplitude, there was a significant main effect of category (*F* = 27.57, *p* < 0.01). The mean amplitude for body responses was larger than the mean amplitude for blob responses. The average peak latency for body responses was 138 ms, but there were no significant differences in latency between blob and body responses.

#### T185 response

The T185 component was most prominent at occipito-temporal sites. The average peak latency for this response was 185 ms for body responses and 192 ms for blob responses. There was a significant main effect of category on the latency of T185 (*F* = 110.07 = 0.866, *p* < 0.001), but not for any other main effect or interaction with latency.

A significant main effect of category was observed for the amplitude of the T185 component (*F* = 67.07, *p* < 0.001), and the mean amplitude was larger when blob stimuli were perceived as a “body.” A significant main effect of hemisphere also was observed, which indicated the mean amplitude was larger in the right hemisphere than in the left hemisphere. In addition, a significant interaction was observed between category and hemisphere (*F* = 12.64, *p* < 0.05). In terms of body responses, the amplitude of the T185 component was larger in the right hemisphere than in the left hemisphere (*p* < 0.001). No inter-hemispheric difference was observed for blob responses.

### Contour maps and source locations

Figure 2C depicts representative contour maps of peak latencies for body and blob responses for one subject. As the figure indicates, responses with latencies around 115 ms were associated with both body and blob responses, and were recorded from the occipital area. Responses with latencies around 135 ms were associated with both response types, and were recorded from the occipito-temporal and occipito-parietal areas. Body responses, however, were recorded from the frontal areas as well. Finally, responses with latencies around 185 ms were associated with both response types and were recorded from the occipito-temporal area, whereas body responses were recorded from regions anterior to those for blob responses.

**Figure 5:**
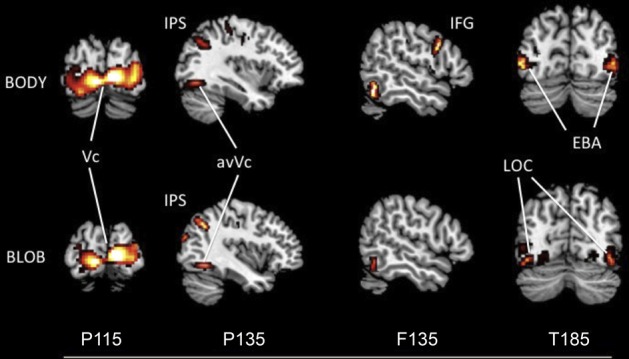
**Statistical parametric maps rendered onto canonical anatomical sections showing the *t* statistic for the “body” (upper) and “blob” (bottom) at each peak latency of P115, P135/F135, and T185 (Vc, visual cortex; anVc, anterior ventral visual cortex; IPS, intraparietal sulcus; LOC, lateral occipital complex; IFG, inferior frontal gyrus; EBA, extrastriate body area)**.

The source locations were analyzed for each component by using an MSP method to highlight areas of overlapping cortical activity. We estimated sources for body responses in the early visual cortex bilaterally (probably the V1/V2) for P115 (18 −94 1; −10 −95 1), the anterior ventral visual cortex (avVc) (40 −78 −15; −40 −78 −10) and intraparietal sulcus (IPS) for P135 (24 −64 61; −29 −64 53), the inferior frontal gyrus (IFG) for F135 (57 14 19; −54 13 11), and the EBA bilaterally for T185 responses (50 −67 5; −49 −69 10; Figure 5). We estimated the sources for blob responses in the early visual cortex bilaterally for P115 (16 −91 5; −15 −95 3), the avVc for P135 (38 −75 −15; −40 −77 −10), and the ventral visual cortex (likely the lateral occipital complex, LOC) for T185 (47 −71 −4; −43 −72 −2; Figure 5). We observed no significant differences in the estimated sources among the visual areas for P115 and P135 body or blob responses (paired *t*-test). However, we observed a significant difference in the estimated source for the IFG with F135 blob and body responses. We observed a significant difference in the estimated source for the EBA (body) and LOC (blob) for T185 responses (*p* < 0.001).

## Discussion

The aim of this study was to determine the electrophysiological correlates underlying disambiguation of blobs when prior instructions are provided. Stimuli were meaningless blobs and were presented to participants with one of two possible instructions. In the HB condition, participants were instructed to anticipate blobs that appear as bodies more frequently than they did in the LB condition. The behavioral results showed that blob stimuli presented with the HB instruction were perceived as a body with a significantly higher probability than the same blob stimuli presented with an LB instruction. This indicates that instructions given to an individual can alter his/her perception significantly, even though the stimuli were identical across conditions, and result in disambiguating ambiguous information. This effect may occur through top-down modulation by producing expectations of an upcoming stimulus. Prior information was thought to play a role in previous contextual modulation and perceptual closure studies, (Cox et al., 2004; Eger et al., 2007). However, these results also suggest that different stimulus interpretations can result when different prior information is given to an individual. This kind of flexibility in perception must help us not only with perceiving and interpreting the ever-changing external world but also with appreciating great ambiguity in art works.

### P115 generated from V1/V2 activity

Significant MEG components peaking around 115 ms were recorded for both body and blob responses in posterior regions, particularly the V1/V2 areas. P115, which is sometimes called P1/M1, is related to the initial processing of visual stimuli and reflects primary visual cortex activation (e.g., Okazaki et al., 2008). Previous studies have shown that the amplitude and latency of the P1/M1 component is not modulated by the stimulus category (Thierry et al., 2006), top-down attention (Lueschow et al., 2004), or an inversion effect of faces (Latinus and Taylor, 2005). P1/M1 amplitude is considered sensitive to variations in low-level features, such as luminance and stimuli contrast (e.g., Itier and Taylor, 2004). The two conditions used in this study were provided with identical sets of stimuli; thus, there were no significant differences in amplitude and latency for P115.

### P135 and F135 generated from the occipito-temporal/parietal and frontal areas

A significant MEG component was recorded from the posterior-temporal/posterior-parietal areas that peaked at approximately 135 ms. Both body and blob P135 responses were generated from the avVc and the IPS. We observed no significant differences in amplitude and latency between P135 body and blob responses. Thus, the V1/V2, avVc, and IPS are active for both percepts, and show similar latencies and amplitudes. The common activity in V1/V2 and the avVc may reflect stimulus-driven processing of visual inputs (Okazaki et al., 2008), which refers to “bottom-up” processing in visual perception (Mechelli et al., 2004).

An interesting finding for this component was activation within the IPS. The IPS is active during imagery (Ishai et al., 2002) and visual matching tasks in which subjects match a target stimulus to a previously presented stimulus (Schendan and Stern, 2008). One recent functional MRI (fMRI) study reported that the IPS was co-activated with the extrastriate cortex during imagery of human bodies (Blanke et al., 2010). This parietal region is thought to be active during imagery regardless of its content (Mechelli et al., 2004). It is possible the current task required body imagery by having to match a stimulus figure presented on the screen to an image in one's mind. Thus, IPS activation observed in this study may reflect such mental imagery and matching processes.

Another interesting observation was that a significantly greater response was elicited for body responses compared to blob responses for the F135 response recorded from the IFG. This finding demonstrates that the IFG participates in the perception of meaningless blobs as a body figures. This pattern of IFG activation was observed only for the body condition and preceded the T185 response. The T185 response is considered the N1 component in ERP studies (Thierry et al., 2006), and has been described as the main “body-selective” component (Thierry et al., 2006; Ishizu et al., 2010).

Prior knowledge may act to modulate the observer's perception by producing expectations for anticipated stimuli in a top-down manner where the perception can be altered from one interpretation to another (Bar, 2004). The IFG and the inferior frontal junction have been associated with expectations that increase the efficiency of perceptual processing of complex objects (Bollinger et al., 2010). A recent study showed that these frontal regions functionally connect to visual cortex in a top-down manner (Pennick and Kana, 2011). Taken together, these findings indicate that IFG activity for perceiving body figures could be linked to a top-down modulation of expectation in a network of regions associated with disambiguation. Expectation of a particular category of objects may include generating visual images and the concepts for those objects. Consistent with this idea, recent studies have demonstrated that the IFG plays a role in processing abstract concepts (Wang et al., 2010), as well as imagining objects, such as faces (Ishai et al., 2002). Thus, creating the perception of a body figure from a meaningless collection of blobs likely requires previous knowledge of the body concept and the ability to imagine the shape. The current study provides evidence that IFG activation correlates with creating expectations that result in stimuli disambiguation, even when no actual physical difference exists across conditions. With respect to the latency of F135, this finding supports a top-down process originating in the frontal cortex. The latency of F135 was even earlier than that of the main category-selective component, T185. A previous MEG study that used the oddball task found that IFG activation was observed at a very early stage and occurred at less than 115 ms (Shtyrov and Pulvermuller, 2007). Other studies using the dynamic causal modeling (DCM) method to reveal brain activation patterns among regions during visual imagery showed that a combined frontal and parietal region activation was followed by activation in category-specific visual areas (Mechelli et al., 2004). In “Mooney” face studies, when subjects perceive a stimulus as a face, activity in the parietal region is enhanced (Dolan et al., 1997) and activity in the ventral visual areas including LOC may be modulated by top-down interpretation (Hsieh et al., 2010). These previous findings suggest a top-down or backward connection from the frontal and parietal regions to category-specific visual cortex (Ishai, 2010). The current results indicated the latency corresponding to activity in the frontal and parietal cortex, as well as specialized visual areas, indicates a similar top-down system may come into play when prior knowledge affects the observer's perception of meaningless visual inputs.

### T185 generated from occipito-temporal areas

Both body and blob responses showed a significant MEG component at posterior and temporal sites that peaked around 185 ms; however, there was a striking difference in the source locations for the T185 components. Activity induced by body responses were localized bilaterally around the EBA in the middle temporal area, a known body-selective region (Thierry et al., 2006; Ishizu et al., 2010), whereas activity induced by blob responses were localized to more posterior and inferior regions in the occipital area that likely included the LOC, a known object-selective region (Malach et al., 1995). Previous fMRI studies that examined the perception of ambiguous stimuli reported the relevant visual processing areas, such as the fusiform face area (FFA) (Sergent and Signoret, 1992; Kanwisher et al., 1997) for face perception, showed greater activation when subjects perceived a stimulus as a meaningful object. This demonstrates that the activation in the FFA is tightly linked to conscious perception of faces (Andrews and Schluppeck, 2004; McKeeff and Tong, 2007). A recent study also showed that imagery of human bodies induces EBA activation even without visual input (Blanke et al., 2010). Consistent with these previous results, the findings of the current study reveal that the T185 component was larger when participants perceived bodies than when they perceived blobs, even though the stimuli were identical. Therefore, the T185 component generated from the EBA is tightly wired to the subjective perception of bodies and is important for the subject's overt perception. Furthermore, this suggests that the subjective percept of blobs is linked to activity within the LOC. Collectively, the EBA shows a strong relationship with the subjective perception of bodies. Indeed, EBA activity is increased when participants think they see a body figure that is physically just a collection of blobs.

Activation within these visual areas was subsequent to those in the frontal and inferior-parietal areas. Previous fMRI studies, and DCM studies mentioned below, suggested that category expectation influences both stimulus-evoked and baseline activity in the visual areas, and these modulations may be driven by a fronto-parietal attentional control network (e.g., Puri et al., 2009). All of these results indicate that prior knowledge modifies brain activity and the final percept to a given stimulus; however, the time course of actual activations in the brain has been largely unaddressed. Our results clearly showed the temporal dynamics between the fronto-parietal and visual areas induced by prior knowledge.

### Neural networks underlie disambiguation

The present study reveals the time course of neural activity underlying disambiguation of ambiguous stimuli with prior knowledge. The order of activation for the body percept began with V1/V2, proceeded to the avVc/IPS and IFG, and ended with the EBA. The order of activation for the blob percept was similar to that for the body and began in V1/V2, proceeded to the avVc/IPS, but ended in the LOC. The entire activation sequence occurred between 115 and 190 ms for both percepts. Collectively, these results reveal the neural system is engaged when the brain interprets meaningless blobs into meaningful information, such as a body figure.

**Figure 6:**
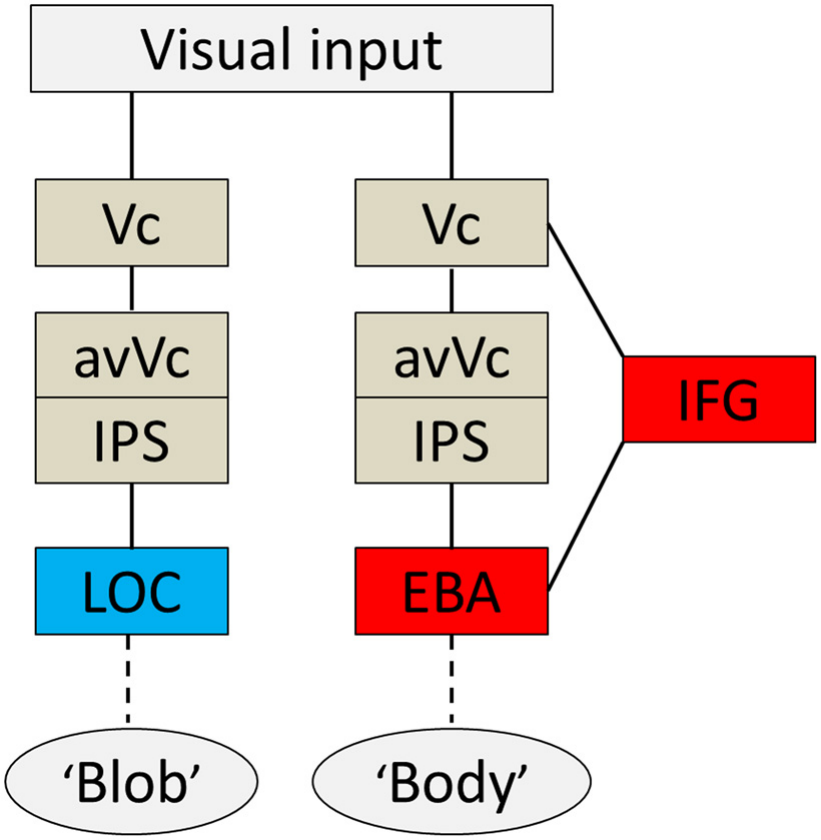
**Summary diagram.** A proposed hypothetical scheme to illustrate the brain systems involved in disambiguation of ambiguous figures, without implying that the two systems are not linked, either directly or indirectly. The system to the left [early visual cortex (Vc), anterior ventral visual cortex (avVc), intraparietal sulcus (IPS), and lateral occipital complex (LOC)] is engaged in “blob” perception, whereas that to the right [Vc, avVc, IPS, inferior frontal gyrus (IFG), and extrastriate body area (EBA)] is engaged in “body” perception.

Based on the present findings and those from previous studies, I propose a hypothetical model of a cognitive system underlying disambiguation of ambiguous stimuli that occurs in two stages. Firstly, preceding EBA activation, the IFG and IPS are active when an observer's percept is altered through prior knowledge. Then, EBA activation comes in; this process is tightly wired to the conscious perception of bodies even without the physical features of real bodies. A diagram of brain areas activated when the subjects subjectively perceived a body or a blob is shown in Figure 6. The second point is perhaps the most important. Theorists have suggested that prior information acts to alter our perceptions in a top-down manner (Bar, 2004), and neuroimaging studies using the DCM method also highlight the importance of early activation in frontal/parietal areas and top-down signals from those areas involved in object recognition (Bar, 2004; Noppeney et al., 2006). Taken together, our findings, along with those of previous studies, suggest that top-down processes originating in IFG and IPS, including imagery and concept forming, play a role in the alteration of percept. Moreover, activity within the IFG associated with expectations is especially important for disambiguating ambiguous information. Furthermore, it implies that both processes are important for creating an interpretation of what people see before the information is sent to the relevant functionally specialized visual areas, as is the case with the EBA or the LOC. However, the neural pathways through which the signal reaches the IFG remain unknown.

### Disambiguation and physical reality

Creativity has yet to be objectively defined. It is difficult to implement common creativity tasks into neuroimaging experiments in an amenable way (Abraham et al., 2012). I acknowledge that other factors contribute to creative processes and studying the full extent of the creative processes requires different experimental paradigms. In this initial study, the ability to disambiguate ambiguous information by producing expectations with prior knowledge is considered one form of creativity, and I have attempted to determine the neural mechanisms underlying disambiguation.

Disambiguation investigated in this study was caused by prior knowledge about upcoming ambiguous stimuli. Previous psychological studies have shown that prior knowledge modulates a range of perception and cognition: color perception, emotion judgment on faces, and interpretation of stories (Hering, 1964/1878; Herr, 1989; Loftus, 1997; Mobbs et al., 2006). Prior knowledge can also act to facilitate our ability to recognize stimuli otherwise uninterpretable (Cox et al., 2004; Bar et al., 2006). As I have argued and previous studies have shown, prior information plays an important role in extracting meaning from what may be otherwise considered meaningless. The findings from the current study revealed the brain regions that correlate with this psychological process.

We do not see the external world as it really is, rather we see it as we think it should be depending on available information. The lights of stars can be turned into myths, the shadows in the darkness can represent the world of death, and meaningless blobs can change into humans. There is no doubt that disambiguation influences human activities and enriches our sensory experiences, and the mechanisms that transform physical reality into something else may be based on neurobiological processes.

### Conflict of interest statement

The author declares that the research was conducted in the absence of any commercial or financial relationships that could be construed as a potential conflict of interest.
